# Impact of Mediterranean Diet Food Choices and Physical Activity on Serum Metabolic Profile in Healthy Adolescents: Findings from the DIMENU Project

**DOI:** 10.3390/nu14040881

**Published:** 2022-02-19

**Authors:** Fabrizio Ceraudo, Giovanna Caparello, Angelo Galluccio, Ennio Avolio, Giuseppina Augimeri, Daniela De Rose, Adele Vivacqua, Catia Morelli, Ines Barone, Stefania Catalano, Cinzia Giordano, Diego Sisci, Daniela Bonofiglio

**Affiliations:** 1 Department of Pharmacy, Health and Nutritional Sciences, University of Calabria, Rende, 87036 Cosenza, Italy; fabrizio.cer96@gmail.com (F.C.); giuseppina.augimeri@unical.it (G.A.); daniela.derose@unical.it (D.D.R.); adele.vivacqua@unical.it (A.V.); catia.morelli@unical.it (C.M.); ines.barone@unical.it (I.B.); stefcatalano@libero.it (S.C.); cinzia.giordano@unical.it (C.G.); diego.sisci@unical.it (D.S.); 2 Health Center srl, 87100 Cosenza, Italy; caparello.giovanna@gmail.com (G.C.); angelo.galluccio@yahoo.it (A.G.); ennioavolio@libero.it (E.A.); 3 Department of Clinical and Experimental Medicine, University Magna Graecia, 88100 Catanzaro, Italy; 4 Centro Sanitario, University of Calabria, 87036 Rende, Italy

**Keywords:** Mediterranean diet, adolescence, physical activity, metabolic profile

## Abstract

Adolescent nutrition and healthy dietary patterns, particularly the Mediterranean diet (MD), have been associated with improved health status and decreased risk of various chronic and metabolic diseases later in life. The aim of this study was to evaluate the impact of the Mediterranean food choices on lipid and glycemic metabolic profile in the total population and in adolescents grouped according to their physical activity (PA) levels at the time of recruitment (T0) and after six months from the administration of a personalized Mediterranean meal plan (T1). As part of the DIMENU study, 85 adolescents underwent measurements of lipid and glucose metabolic profile at T0 and T1. Using three positive items from KIDMED test related to the consumption of typical Mediterranean food (olive oil, fish, and nuts) and three negative items on dietary habits (going to fast-food, consuming biscuits, and candies), we categorized adolescents into six sets in which biochemical parameters were analyzed. In the total sample, significant reductions in serum total cholesterol, LDL, and glucose concentrations were observed for all the sets over the study period. Notably, active subjects, who had a better serum metabolic profile, showed significant improvements of glycemic control after 6 month follow up, while in sedentary adolescents and in those performing moderate PA significant reduction in glycemia, total cholesterol, and LDL was found in all sets. In conclusion, adopting the typical Mediterranean food choices led to a significant reduction in glucose and lipid profile in healthy adolescents, thus making the MD and PA a winning combination for health status.

## 1. Introduction

The Mediterranean diet (MD) represents a nutritional model inspired by typical culinary traditions of populations bordering the Mediterranean basin, which share the same food availability. The identification of this geographical area is based on several scientific and epidemiological evidence demonstrated by Ancel Keys during the second half of the last century in the “Seven Country Study” [1]. Nowadays, the MD is considered a gold standard of healthy eating as far as it is related to greater longevity and improvement in health and quality of life [2], as well as a reduction of the risk of cardiovascular disease (CVDs), cancer, obesity, diabetes, osteoporosis, and cognitive illnesses [3,4,5,6,7,8,9]. Benefits of the MD are due to the daily consumption of whole grains, in association with legumes, fruits, and vegetables as sources of vitamins and minerals, as well as low glycemic index carbohydrates and fiber, which can slow down the digestion of starch and the absorption of sugars with a lower increase in blood glucose over the postprandial period, improving insulin sensitivity and reducing the cholesterol absorption [10]. Soluble fiber, thanks to its ability to bind bile acids, also stimulates the growth of the gut microbiome (prebiotic effect), which ferments fibers with the production of short chain fatty acids, such as acetic, butyric, and propionic acids, and they are able to modulate the gluconeogenesis and lipogenesis in the liver and limit the production of potentially carcinogenic substances [11]. The daily consumption of extra virgin olive oil along with additional consumption of nuts are sources of antioxidant molecules, such as tocopherol (vitamin E), and unsaturated fatty acids (oleic and linoleic acids) that reduce the LDL cholesterol without affecting HDL [12,13]. It also involves moderate consumption of fish, the source of high biological value proteins and omega-3 fatty acids, which perform an anti-inflammatory function and a triglyceride-lowering activity, interfering with triglyceride incorporation in VLDL at hepatic level [14]. Regular but moderate consumption of wine, generally during meals, provides good amounts of polyphenols, including resveratrol, known for its antioxidant and anti-inflammatory effects, partly due to the ability to reduce cyclooxygenase-2 expression and consequently the prostaglandin production [15,16,17]. Weekly consumption of poultry and dairy products assures great protein sources with a moderate fat content. Occasional consumption of red meat and sweets is associated with a reduced intake of cholesterol and sugar [18,19]. The health effects of this pattern do not derive only from their individual components, but from the synergy between the MD components [20]. MD pattern is not just a way of eating, but it is a real dietary model that involves the total lifestyle and impacts on daily habits thanks to seasonality and biodiversity, accompanied by cultural elements such as conviviality, adequate night’s rest, and physical activity [21].

Therefore, establishing and applying healthy eating behaviors starting from adolescence is important so that they persist into adulthood [22]. They represent the key elements, combined with regular physical activity, associated with a reduction in risk of obesity and in the prevention of metabolic syndrome and chronic diseases [23]. Adolescence is a period of physical growth and rapid development characterized by significant changes in cognitive, physiological, and emotional profile, with effects that can affect the quality of life, well-being, and health of individuals. In the recent decades, the phenomenon of food “westernization” has been described as the consumption of foods rich in refined carbohydrates, saturated fats, salt, and proteins (chips, salty snacks, fast food, and candies) and soft drinks. In addition, the sedentary lifestyle induced by modern society caused by automated equipment, motorized transport, and the increased time spent watching television is responsible for the increased incidence of obesity as well as metabolic and chronic non-communicable diseases [24]. 

The aim of this study was to evaluate the impact of the MD food choices on lipid and glycemic metabolic profile in the total population and in adolescents grouped according to their physical activity (PA) levels at the time of recruitment (T0) and after six months from the administration of a personalized Mediterranean meal plan (T1).

## 2. Materials and Methods

### 2.1. Study Population

The adolescents were recruited into the DIMENU research project (DIeta MEditerranea e NUoto, FESR-FSE 2014–2020. Prot 52243/2017) enrolled by the Castrolibero Institute of Education (Cosenza, Italy) and by sports associations of the Calabria region, Italy [25,26,27,28]. The total population studied is composed of 85 subjects (44 girls and 41 boys) aged between 14 and 17 years. Criteria of exclusion from the study included health problems, drug use, and any type of restrictive diet (i.e., low calorie, low carbohydrate, and low fat content). The participants did not have any kind of cognitive or physical/motor limitation. The adolescents participating in the study provided verbal informed consent, while their parents signed a written informed consent form to allow their participation. The rationale of the research project and the adequacy of the protocol were approved by the Ethics Committee of the University of Calabria, Italy (# 5727/2018).

### 2.2. Nutritional History

The assessment of the participants’ nutritional status and medical history was made during an interview with a team of professionals (endocrinologists and biologist nutritionists). During the interview, the students provided the anamnestic data (general data, medical history, eating habits, and intensity of physical activity). After that, participants were divided into three groups, on the basis of their difference in the physical activity levels, expressed in MET (metabolic-equivalent unit expressed as 1 kcal/kg/hour), in accordance with recommendations issued by the WHO [29]. Thus, the groups obtained were: (1) Group A, physical inactivity, less than 3 METs; (2) Group B, moderate physical activity, between 3 and 6 METs (cycling, dancing, brisk walking, gymnastics, ballet, water aerobics, recreational swimming) for at least 60 min a day; (3) Group C, vigorous physical activity, above 6 METs (jogging or running, boxing, tennis, football, basketball, squash, swimming, aerobic dance and volleyball) for at least 60 min per day.

### 2.3. Mediterranean Diet Adherence Test (KIDMED)

**Figure 1:**
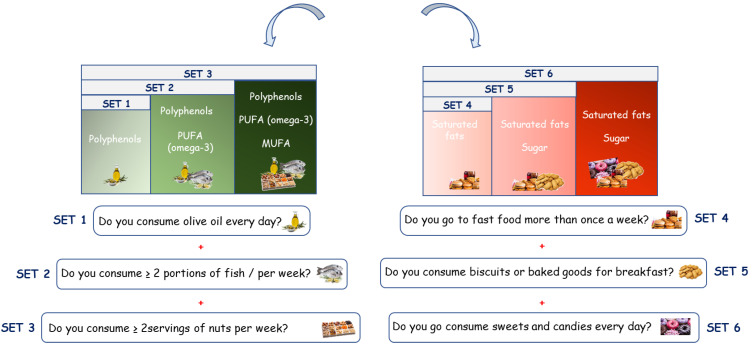
Classification of the Mediterranean Diet food choices according to selected questions with positive (sets 1, 2 and 3) or negative (sets 4, 5, and 6) impact on MD adherence.

The KIDMED test (Mediterranean Diet Quality Index for children and teenagers) has been used to assess adherence to Mediterranean dietary patterns in children and adolescents [30], updated on the new food pyramid of the International Foundation of MD, as previously reported [26]. The questionnaire is made up of 16 questions, of which 4 denote a negative connotation and 12 questions denote a positive connotation to MD. To evaluate the impact of the Mediterranean food choices on the different parameters analyzed at T0 and T1, we selected questions with positive connotations from KIDMED test, which were categorized in the following sets: set 1: “Do you consume olive oil every day?”; set 2: “Do you consume olive oil every day?” and “Do you consume ≥ 2 portions of fish/per week?”; set 3: “Do you consume olive oil every day?”, “Do you consume ≥ 2 portions of fish/per week?”, and “Do you consume ≥ 2 servings of nuts per week?”. Moreover, on the basis of negative items impacting the adherence to the MD from KIDMED test, we categorized three sets: set 4:”Do you go to fast-food restaurants more than once a week?”; set 5: “Do you go to fast-food restaurants more than once a week?” and “Do you consume biscuits or baked goods for breakfast?”; set 6: “Do you go to fast-food restaurants more than once a week?”, “Do you consume biscuits or baked goods for breakfast?”, and “Do you consume sweets and candies every day?” (Figure 1). 

### 2.4. Anthropometric Parameters and Bioelectrical Impedance Analysis

Anthropometric data were collected using a validated protocol [31]. Participants’ weights were determined using the KERN MPC250K100 M scale with a load capacity of 250 kg and an accuracy of 100 g. Height was determined using a Seca stadiometer with a maximum capacity of 220 cm and an accuracy of 1 mm. The body circumferences of each participant were measured using a validated ergonomic tape Seca 201, with a measuring range from 1 to 205 cm and a division of 1 mm. Body mass index (BMI) was calculated by dividing the body weight in kilograms by the square of height in meters [BMI = weight (kg)/height^2^ (m)]. The BMI z-score was calculated on the basis of the World Health Organization data [BMI z-score = [(BMI/M(t))L(t) − 1]/L(t)S(t)]. In particular, waist and hip circumferences were used to calculate the waist/hip ratio (WHR). Body composition assessment was performed after a 12 h overnight fast according to the measurement protocol using bioimpedentiometric analysis (BIA) (single-frequency 50 kHz BIA 101 S, Akern-Systems, Florence, Italy). Each subject underwent BIA, performed to evaluate resistance, reactance, phase angle (PhA), total body water (TBW), body cell mass (BCM), fat-free mass (FFM), and fat mass (FM). Data were analyzed using Bodygram Plus software Version 1.2.2.8. (Akern Srl; Florence, Italy). Measurements and assessments of bioelectrical parameters were made at recruitment time (T0) and after six months (T1). 

### 2.5. Biochemical Measurements

Venous blood samples were collected after 8–10 h of overnight fasting, both at baseline (T0) and T1. The serum was obtained after centrifugation at 3000 rpm for 10 min and stored in sterile tubes at 4 °C for no more than 4 h during the morning of collection. The biochemical parameters were determined by a Konelab 20i chemistry analyzer (Thermo Electron Corporation, Vantaa, Finland) according to standard procedures. Subsequently, the serum samples were stored at −80 °C. Serum insulin levels were measured with an enzyme-linked immunosorbent assay (Novatec Immundiagnostica GmbH, Dietzenbach, Germany) following the manufacturer’s instructions. The lowest detectable insulin concentration was 0.25 μIU/mL at a 95% confidence limit; the intra-assay variability was within ≤5%. 

### 2.6. Mediterranean Personalized Food Plan

Each participant received a personalized Mediterranean plan that was based on their nutritional status and different levels of physical activity (PA). Throughout the program period, nutritionists provided participants with indications on the choice of typical Mediterranean foods. The dietary approach is based on the latest guidelines of the MD as previously reported [25]. The dietary scheme provided 15–20% of calories through protein, 45–60% of calories through carbohydrates, and 25–30% of calories through fat, with the redistribution of macro- and micronutrients according to the different daily energy expenditure (TDEE) of each subject as recommended by the Italian Society of Human Nutrition [32]. Moreover, we have to underline the fact that distribution of some macronutrients such us protein (range from 1 to 1.5 g/day) were also customized according to physical activity (PA) level (sedentary, moderately active, and vigorous), and thus the same diet plan was not provided to everyone. The foods included in the diet are obviously typically Mediterranean ones, and meals included an abundance of plant food (fruits, vegetables, whole grains, nuts, and legumes); low-fat dairy products, fish, poultry, and eggs in moderate amounts; olive oil as the primary source of fat; and low consumption of red meats, processed foods, and saturated lipids. Meals and food plans were designed using MetaDieta software version 4.2.1. (Meteda S.r.l, Roma, Italy). 

### 2.7. Statistical Analysis

All statistical analyses were performed using SigmaPlot Version 12.0 (Systat, San Jose, CA, USA). A Kolmogorov–Smirnov test (with Lilliefors’ correction) was used to verify data normality. Data were reported as the mean and standard deviation (SD), and statistical differences between samples were evaluated by using parametric tests (one-way ANOVA and Student’s *t*-test). Statistical significance was set at *p* < 0.05.

## 3. Results

### 3.1. General Characteristics and Metabolic Profile of the Study Population

Anthropometric and bioimpedance measurements as well as metabolic parameters were evaluated in the total sample of adolescents (*n* = 85) before they started the food plan (T0) and 6 months after (T1) as reported in Table 1. From the comparison of the total sample in the two observation periods, there were no significant changes, except for the BMI, which was increased at T1 (*p* = 0.0237), remaining within the range of normal values. Interestingly, after 6 months, a significant reduction in fasting blood glucose (*p* = 0.0001), total cholesterol (*p* = 0.0002), and LDL (*p* = 0.0009) was found.

### 3.2. Impact of the Mediterranean Diet Food Choices on the Adolescent Metabolic Profile

Having previously evaluated that in this population the KIDMED score at T1 compared with T0 were significantly increased after 6 month follow-up (T0 = 6.04 ± 2.34 vs. T1 = 6.94 ± 2, *p* = 0.006) [25], in order to deeper investigate the contribution of the MD food choices in the improvement of metabolic profile, we selected questions from the KIDMED test relating to the consumption of foods affecting lipid profile (total cholesterol, LDL, HDL, and triglycerides) and glucose homeostasis (glucose and insulin). Table 2 shows the changes in the metabolic profile of the participants categorized into three sets, which refer to some of the questions chosen from the KIDMED test as follows: (1) “Do you consume olive oil every day?”; (2) “Do you consume olive oil every day?” and “Do you consume ≥2 portions of fish/per week?”; (3) “Do you consume olive oil every day?”, “Do you consume ≥2 portions of fish/per week?”, and “Do you consume ≥2 servings of nuts per week?”. Analyzing the biochemical parameters in the sets identified, at both times of observation, we found that particular significance emerged. Interestingly, we observed significant improvements in fasting blood glucose in all the sets (*p* = 0.0001 in set 1, *p* = 0.0001 in set 2, and *p* = 0.0019 in set 3). Total cholesterol and LDL concentrations significantly decreased in set 1 (*p* = 0.0004 and *p* = 0.0012, respectively), in set 2 (*p* = 0.0173 and *p* = 0.0123, respectively), and in set 3 (*p* = 0.0022 and *p* = 0.0086, respectively) (Table 2).

Moreover, questions from KIDMED test relating to improper eating habits were used to categorize adolescents that gave a negative answer to the questions in sets 4, 5, and 6, as described: (4)”Do you go to fast-food restaurants more than once a week?”; (5) “Do you go to fast-food more than once a week?” and “Do you consume biscuits or baked goods for breakfast?”; (6) “Do you go to fast-food more than once a week?”, “Do you consume biscuits or baked goods for breakfast?”, and “Do you consume sweets and candies every day?”. Notably, total cholesterol and LDL levels significantly decreased in set 4 (*p* = 0.0011 and *p* = 0.0047, respectively), in set 5 (*p* = 0.0026 and *p* = 0.0226, respectively), and in set 6 (*p* = 0.0049 and *p* = 0.0334, respectively). Furthermore, there was a drastic reduction of fasting glycaemia in all the sets (*p* = 0.0001 in set 4, *p* = 0.0001 in set 5, and *p* = 0.0024 in set 6) (Table 3).

### 3.3. Impact of the Mediterranean Diet Food Choices on Metabolic Profile in the Adolescents Grouped According to the Different Physical Activity Levels

On the basis of the self-reported PA intensity levels, we grouped our adolescents into the physical inactivity (Group A; *n* = 23), moderate-intensity PA (Group B; *n* = 34), and vigorous-intensity PA (Group C; *n* = 28) levels, which were confirmed by interview over the study period [25]. We have previously reported that the MD adherence increased particularly in adolescents performing moderate (KIDMED score at T0: 5.57 ± 2.30 and at T1: 6.94 ± 1.66) and vigorous (KIDMED score at T0: 6.30 ± 2.16 and at T1: 7.61 ± 1.77) PA levels compared to sedentary (KIDMED score at T0: 5.96 ± 2.10 and at T1: 6.61 ± 2.35) [14]. Here, we analyzed, in the three PA groups of adolescents, the intra- and inter-group differences in the metabolic profile at T0 and T1 (Table 4). From the intragroup analysis, we found a significant reduction in fasting blood glucose (*p* = 0.0052), total cholesterol (*p* = 0.0008), and LDL (*p* = 0.0028) in Group A. Adolescents from Group B showed total cholesterol and LDL levels that were significantly reduced (*p* = 0.0001 and *p* = 0.0152, respectively). In Group C, subjects had significant reductions in fasting glucose (*p* = 0.0001). Table 4 also shows the intergroup comparison (A vs. B, A vs. C, and B vs. C) at T0 and T1, performed by using ANOVA test. At T0, Group C had statistically higher fasting blood glucose values (*p* = 0.0309) compared to Group B, while plasma LDL concentrations were statistically lower in Groups B and C than in Group A (*p* = 0.0488 and *p* = 0.0015, respectively). Insulinemia was lower in Group B than in Group A (*p* = 0.0277) at T0, while both Groups B and C had significantly lower values than Group A (*p* = 0.0009 and *p* = 0.0001, respectively) at T1.

In addition, the impact of specific MD food choices over time (T0 vs. T1) was also observed for the three groups on the basis of the PA performed (Table 5). The results in Group A demonstrate a significant decrease in total cholesterol and LDL in set 1 (*p* = 0.0032 and *p* = 0.0057, respectively) and in fasting blood glucose (*p* = 0.0019). In set 2, significant reductions were recorded for total cholesterol (*p* = 0.0328) and blood glucose (*p* = 0.0070). In Group B, a significant reduction in total cholesterol and LDL was present in set 1 (*p* = 0.0135 and *p* = 0.0059, respectively) and in glucose levels in set 2 (*p* = 0.0392). Regarding Group C, in all sets, fasting glucose was significantly reduced (*p* = 0.0001 in set 1, *p* = 0.0001 in set 2, *p* = 0.0019 in set 3). No significant changes were found in these sets for both anthropometric and bioimpedance parameters (data not shown).

Differences between T0 and T1 were also performed in the three groups on the basis of PA (A, B, and C) (Table 6). Again, significant differences emerged regarding the lipid metabolic profile and glucose concentrations. Adolescents from Group A had a reduction in total cholesterol and LDL in all three sets (*p* = 0.0014 and *p* = 0.0052, in set 4; *p* = 0.0066 and *p* = 0.0257 in set 5; *p* = 0.0024 and *p* = 0.0251 in set 6), while fasting glucose was significantly reduced in sets 4 and 6 (*p* = 0.0052 and *p* = 0.0034, respectively). Moreover, adolescents from Group B showed significant changes in total cholesterol and LDL in set 4 (*p* = 0.0454 and *p* = 0.0437, respectively) and in set 6 (*p* = 0.0264 and 0.0473, respectively), and changes in total cholesterol in set 5 (*p* = 0.0406). Interestingly, in Group C, subjects had significant reductions in fasting blood glucose in the three sets (*p* = 0.0001 in set 4, *p* = 0.0001 in set 5, and *p* = 0.0054 in set 6), along with a reduction in insulinemia in sets 4 and 5 (*p* = 0.0274 and *p* = 0.0338, respectively). No significant differences emerged with regards to the anthropometric and bioimpedance parameters (data not shown).

## 4. Discussion

In this study, we evaluated the impact of different MD food choices on serum metabolic parameters in a population of healthy adolescents performing different PA levels. Nowadays, the importance of optimal adherence to the MD and PA is widely known as the main tool in countering the onset of chronic non-communicable diseases, often associated with increased consumption of unhealthy foods (junk food) and an increasingly sedentary lifestyle. For this reason, it is necessary to promote the Mediterranean pattern, especially during adolescence, in order to educate the new generations to have good eating habits that will result in the maintenance of good health and a long life expectancy. Globally, adolescents show a poor adherence to nutritional recommendations, preferring an excess of energy coming from fats at the expense of that taken from carbohydrates and proteins; in addition, there is a low consumption of those foods that characterize MD such as fruits, vegetables, legumes, and fish, along with a widespread habit of skipping breakfast [25,26,28]. The daily consumption of sweets and sugary drinks affects a non-negligible share of adolescents [33]. In our population, the adherence to the MD was evaluated by the KIDMED test, which showed an increased score over the study period. We found a significant difference only for the BMI values, but not for the BMI z-score, in the follow-up study, although, in presence of unchanged other anthropometric and bioimpedance measurements, BMI was not the diagnostic measure for adiposity in adolescents. Conversely, it has been reported that BMI and waist circumference could be considered diagnostic tests for fatness, while WHR is less useful in adolescents [34]. Interestingly, we observed a significant reduction in the serum levels of glucose as well as total cholesterol and LDL in the total sample of adolescents, indicating that the promotion of MD pattern and the increased MD adherence had beneficial effects on serum metabolic profile. Specifically, when we categorized adolescents on the basis of specific MD food choices, we observed over the study period significant decreased levels of total cholesterol, LDL, and glycemia in the sets of participants who consumed “extra virgin olive oil every day” alone (set 1) or together with “two or more portions of fish per week” (set 2) and in combination with “two or more servings of nuts per week” (set 3). These questions referred to the intake of typical recommended MD foods that are rich with monounsaturated fats (MUFA), polyphenols, and polyunsaturated fatty acids (PUFA) including omega-3-fatty acids, representing essential Mediterranean diet components. Similarly, in the sets of participants who “don’t go to fast-food restaurants more than once a week” alone (set 4) or in combination with “don’t consume biscuits or baked goods for breakfast” (set 5) or with “don’t consume sweets and candies every day” (set 6), we found in the follow-up reduced concentrations of serum total cholesterol, LDL, and glycemia. These results fit well with a recent open label study [35], in which Velázquez-López et al. reported that a Mediterranean pattern-based diet improves lipid and glycemic profile in obese children and adolescents. 

Our previous observations showing that PA intensity levels positively influenced healthy dietary pattern [25] represent encouraging results since those who practice vigorous PA began to adopt good eating habits that could last over time. This is in line with other studies [28,36,37] in which the increase in consumption of junk food, sweets, and candies are related to the habits of mainly sedentary adolescents. It was interesting to note that in our population sample, active adolescents display a better metabolic profile compared to sedentary adolescents in terms of LDL and insulin concentrations. This leads us to speculate that the myokines, such as interleukin-6 (IL-6), produced in working skeletal muscle in larger amounts, play a beneficial role in metabolic homeostasis, improving insulin sensitivity and lipolysis and thus making the MD and PA a winning combination for health status. Notably, the blood chemistry parameters significantly improved with the adherence to the specific MD food consumption over the study period in all groups of adolescents from sedentary to moderate/vigorous PA intensity levels. In particular, in sedentary adolescents categorized by positive and negative items of the KIDMED test, we observed a significant reduction in the serum levels of total cholesterol, LDL, and glycemia over the study period, indicating the beneficial role of dietary pattern. A similar trend was found in adolescents who practice moderate PA, while in active subjects, glycemia was significantly reduced in the positive sets, and both glycaemia and insulinemia were decreased in the negative sets after 6-month follow-up. These latter findings fit well with the results from several meta-analyses [38,39,40] that demonstrated how MD guarantees optimal glycemic control when compared with other dietary models.

The limitations of the study include a relatively small sample size, particularly when the total sample was divided into the PA groups, as well as the lack of objective methods to measure PA levels. Adolescence is a phase of life that involves a series of complex alterations at endocrine levels, and this can represent confounding effects on hormonal and metabolic changes occurring during puberty. However, our study strengthens the importance of improving healthy dietary habits and encouraging our adolescents towards better food choices that have been pursued and confirmed in the follow-up. Future studies with a larger number of participants and a different age range (from adolescence to adulthood) and, in particular, the comparison among participants of different age could add considerable insights to the field. 

## 5. Conclusions

In conclusion, findings from this study show that the introduction of a personalized food plan based on MD principles in an adolescent sample led to a significant improvement in glucose and lipid profile in the follow-up, particularly when subjects adopt to the typical MD food choices. This demonstrates the effectiveness of food education programs and how these are implemented and translated into good eating habits in the adolescent population and for the entire lifetime.

## Figures and Tables

**Table 1:** Anthropometric and bioimpedance parameters and metabolic profile in adolescents at T0 and T1.

	T0		T1		
Anthropometric Parameters	Mean	SD	Mean	SD	*p*-Value
Weight (kg)	62.426	12.39	63.518	12.483	0.5685
Height (cm)	165.854	7.818	167.134	8.508	0.3087
BMI (kg/m^2^)	22.685	3.685	23.75	2.252	**0.0237**
BMI z-score	0.48	0.86	0.53	0.76	0.6884
WHR	0.772	0.047	0.781	0.065	0.3024
**Bioimpedentiometric Parameters**					
PhA (°)	6.128	0.693	6.285	0.777	0.1860
BCM (Kg)	26.468	5.714	26.929	5.844	0.6043
FFM (Kg)	48.627	8.987	48.866	9.144	0.8632
FM (Kg)	13.799	7.448	15.291	7.057	0.1826
TBW (%)	36.241	6.587	36.015	6.962	0.8327
**Metabolic Profile**					
Total cholesterol (mg/dL)	155.24	27.7	139.89	24.31	**0.0002**
LDL (mg/dL)	83.4	25.96	71.576	19.02	**0.0009**
HDL (mg/dL)	58.93	14.19	56.76	13.42	0.3071
Triglycerides (mg/dL)	64.39	31.07	57.67	24.43	0.1189
Glucose (mg/dL)	83.46	7.47	77.41	8.48	**0.0001**
Insulin (μIU/mL)	10.35	4.89	11.03	6.1	0.4237

BMI, body mass index; WHR, waist hip ratio; PhA, phase angle; BCM, body cell mass; FFM, fat-free mass; FM, fat mass; TBW, total body water; LDL, low-density lipoprotein; HDL, high-density lipoprotein. Statistical differences were determined by Student’s *t*-test. In bold are reported statistically significant values.

**Table 2:** Comparison between T0 and T1 in the metabolic parameters of adolescents categorized in three sets according to the positive KIDMED items.

KIDMED Items		SET 1			SET 2			SET 3		
Subjects		(78 vs. 78)			(51 vs. 53)			(20 vs. 17)		
Parameters		Mean	SD	*p*-Value	Mean	SD	*p*-Value	Mean	SD	*p*-Value
Total cholesterol (mg/dl)	T0	155.00	27.30	**0.0004**	153.82	28.36	**0.0173**	159.45	26.72	**0.0022**
	T1	140.08	23.92		141.77	22.15		133.35	20.06	
LDL (mg/dl)	T0	83.28	25.56	**0.0012**	82.84	26.26	**0.0123**	89.30	21.22	**0.0086**
	T1	71.44	18.92		72.00	16.12		71.47	17.02	
HDL (mg/dl)	T0	59.04	14.25	0.3782	57.95	15.06	0.9508	57.05	13.44	0.1511
	T1	57.08	13.44		57.77	14.61		50.94	11.57	
Triglycerides (mg/dl)	T0	63.31	27.21	0.1843	65.07	35.33	0.3659	65.15	26.66	0.1691
	T1	57.82	24.11		59.66	24.67		54.47	17.86	
Glucose (mg/dl)	T0	83.46	7.40	**0.0001**	83.84	7.79	**0.0001**	83.84	7.79	**0.0019**
	T1	77.18	8.51		75.79	7.10		75.59	6.97	
Insulin (μIU/mL)	T0	10.42	5.08	0.8056	9.42	4.69	0.3315	9.42	4.69	0.2151
	T1	10.63	5.55		10.26	6.00		12.02	7.69	

LDL, low-density lipoprotein; HDL, high-density lipoprotein. Statistical differences were determined by Student’s *t*-test. In bold are reported statistically significant values.

**Table 3:** Comparison between T0 and T1 in the metabolic parameters of adolescents categorized in three sets according to the negative KIDMED items.

KIDMED Items Subjects		SET 4			SET 5			SET 6		
		(73 vs. 77)			(55 vs. 68)			(30 vs. 31)		
Parameters		Mean	SD	*p*-Value	Mean	SD	*p*-Value	Mean	SD	*p*-Value
Total cholesterol (mg/dl)	T0	153.96	27.28	**0.0011**	154.44	26.08	**0.0026**	155.57	25.92	**0.0049**
	T1	139.87	24.53		140.19	25.13		136.29	25.58	
LDL (mg/dl)	T0	82.47	26.12	**0.0047**	81.40	25.42	**0.0226**	79.40	23.28	**0.0344**
	T1	71.79	18.99		72.07	19.35		67.58	19.22	
HDL (mg/dl)	T0	58.41	14.51	0.4348	60.07	14.95	0.1791	63.07	16.08	0.1012
	T1	56.61	13.65		56.51	14.16		56.13	16.45	
Triglycerides (mg/dl)	T0	65.45	33.15	0.0865	64.80	34.96	0.2076	65.63	27.59	0.7400
	T1	57.23	24.79		57.91	25.26		63.26	28.03	
Glucose (mg/dl)	T0	83.92	7.00	**0.0001**	84.00	6.44	**0.0001**	82.73	6.28	**0.0024**
	T1	77.87	8.47		77.85	8.76		77.13	7.45	
Insulin (μIU/mL)	T0	10.66	4.78	0.9079	10.58	4.95	0.8276	9.76	3.68	0.1587
	T1	10.76	5.99		10.80	6.26		11.60	6.07	

LDL, low-density lipoprotein; HDL, high-density lipoprotein. Statistical differences were determined by Student’s *t*-test. In bold are reported statistically significant values.

**Table 4:** Biochemical and metabolic parameters in adolescents grouped according to different PA levels (Groups A, B, and C) at T0 and T1.

	Group A				Group B				Group C									
	T0		T1		T0		T1		T0		T1		*p*-Value T0			*p*-Value T1		
	Mean	SD	Mean	SD	Mean	SD	Mean	SD	Mean	SD	Mean	SD	A vs. B	A vs. C	B vs. C	A vs. B	A vs. C	B vs. C
Total cholesterol (mg/dl)	164.61	28.51	138.65 *	19.65	154.85	29.25	139.29 *	26.32	148.00	23.46	141.64	25.13	0.3854	0.0835	0.5885	0.9948	0.9021	0.9256
LDL (mg/dl)	97.87	30.08	74.35 *	17.09	82.09	23.26	68.62 *	21.26	73.11	20.19	72.89	16.98	**0.0488**	**0.0015**	0.3238	0.5094	0.9604	0.6557
HDL (mg/dl)	54.65	12.58	52.48	11.98	58.53	15.84	58.88	15.06	62.93	12.58	57.71	11.43	0.5621	0.0955	0.4375	0.1822	0.3470	0.9370
Triglycerides (mg/dl)	60.70	21.10	59.13	21.68	70.50	39.47	59.21	28.13	60.00	25.64	54.61	21.10	0.4741	0.9965	0.3849	>0.9999	0.7914	0.7454
Glucose (mg/dl)	82.61	6.34	76.57 *	7.55	81.62	8.35	77.94	10.18	86.39	6.45	77.46 *	6.60	0.8685	0.1585	**0.0309**	0.823	0.9262	0.9741
Insulin (μIU/mL)	12.43	6.04	15.58	8.00	9.04	4.24	10.04	3.92	10.20	4.07	8.51	4.19	**0.0277**	0.2243	0.6109	**0.0009**	**<0.0001**	0.5181

PA, physical activity; LDL, low-density lipoprotein; HDL, high-density lipoprotein. Statistical differences between T0 and T1 in each PA group were determined by Student’s *t*-test. * *p* < 0.005. Statistically significant differences among Groups A, B, and C at T0 and T1 were calculated by ANOVA test. In bold are reported statistically significant values..

**Table 5:** Biochemical and metabolic parameters in adolescents from Groups A, B and C categorized into three sets according to the positive KIDMED items at T0 and T1.

KIDMED		SET 1			SET 2			SET 3		
**Subject Group A**		**(22 vs. 21)**			**(12 vs. 11)**			**(4 vs. 5)**		
**Parameters**		**Mean**	**SD**	***p*-Value**	**Mean**	**SD**	***p*-Value**	**Mean**	**SD**	***p*-Value**
Total cholesterol (mg/dl)	T0	163.82	28.92	**0.0032**	163.42	30.66	**0.0328**	144.33	20.77	0.3039
	T1	139.90	20.24		137.64	22.35		128.20	22.33	
LDL (mg/dl)	T0	75.19	17.73	**0.0057**	74.73	15.72	0.0737	82.00	24.60	0.6626
	T1	97.36	30.69		96.25	32.92		75.80	16.33	
HDL (mg/dl)	T0	54.18	12.67	0.8071	55.25	13.50	0.4592	48.83	15.59	0.2687
	T1	53.24	12.40		50.82	14.68		39.40	7.57	
Triglycerides (mg/dl)	T0	61.50	21.23	0.5205	60.08	22.17	0.9918	65.83	15.97	0.9176
	T1	57.52	18.90		60.00	13.13		64.80	12.95	
Glucose (mg/dl)	T0	82.95	6.26	**0.0019**	83.17	6.78	**0.0070**	83.17	6.78	0.2497
	T1	75.90	7.63		73.91	8.08		75.80	9.98	
Insulin (μIU/mL)	T0	15.58	8.18	0.6209	15.39	8.73	0.9080	19.45	4.78	0.2151
	T1	14.41	7.15		14.98	9.02		11.03	9.12	
**Subject Group B**		**(30 vs. 31)**			**(20 vs. 22)**			**(5 vs. 4)**		
**Parameters**		**Mean**	**SD**	***p*-Value**	**Mean**	**SD**	***p*-Value**	**Mean**	**SD**	***p*-Value**
Total cholesterol (mg/dl)	T0	155.53	27.93	**0.0135**	151.75	31.21	0.2266	149.00	38.33	0.5062
	T1	137.97	25.89		141.95	18.23		134.250	18.428	
LDL (mg/dl)	T0	83.10	22.35	**0.0059**	82.40	24.55	0.0636	83.80	37.83	0.6351
	T1	67.16	21.24		70.09	16.90		72.750	25.786	
HDL (mg/dl)	T0	58.63	15.83	0.9520	54.90	16.98	0.4341	53.80	11.71	0.6138
	T1	58.87	15.18		58.91	15.91		50.250	7.182	
Triglycerides (mg/dl)	T0	60.03	29.27	0.3411	64.82	29.91	0.4705	56.80	33.84	0.9918
	T1	68.27	32.17		71.20	35.19		57.000	17.010	
Glucose (mg/dl)	T0	81.10	8.01	0.1882	81.50	8.31	**0.0392**	81.50	8.31	0.1367
	T1	77.94	10.34		76.27	7.59		72.75	8.34	
Insulin (μIU/mL)	T0	9.04	4.51	0.3492	7.80	4.56	0.1327	7.80	4.56	0.1610
	T1	10.06	3.92		9.86	4.14		12.55	3.70	
**Subject Group C**		**(26 vs. 26)**			**(19 vs. 20)**			**(11 vs. 8)**		
**Parameters**		**Mean**	**SD**	***p*-Value**	**Mean**	**SD**	***p*-Value**	**Mean**	**SD**	***p*-Value**

Total cholesterol (mg/dl)	T0	146.92	23.48	0.5348	149.58	22.04	0.4678	151.73	19.98	0.1215
	T1	142.73	24.84		143.85	26.41		136.125	21.464	
LDL (mg/dl)	T0	71.58	18.00	0.6898	73.68	17.42	0.8412	73.64	19.13	0.5001
	T1	73.50	16.45		72.60	16.01		68.125	14.066	
HDL (mg/dl)	T0	63.62	12.55	0.1049	64.32	12.86	0.2770	65.36	14.04	0.2492
	T1	58,04	11,80		60,35	12,44		58,56	8.73	
Triglycerides (mg/dl)	T0	59.12	25.50	0.5850	58.32	27.82	0.5363	64.18	32.79	0.1972
	T1	55.42	21.56		53.80	22.80		46.750	19.002	
Glucose (mg/dl)	T0	86.62	6.64	**0.0001**	86.74	7.23	**0.0001**	86.74	7.23	**0.0019**
	T1	77.46	6.60		76.30	6.11		76.88	4.22	
Insulin (μIU/mL)	T0	10.19	4.21	0.1555	10.03	4.49	0.1817	10.03	4.49	0.2151
	T1	8.51	4.19		8.11	4.32		7.10	3.81	

LDL, low-density lipoprotein; HDL, high-density lipoprotein. Group C, vigorous physical activity. Statistical differences were determined by Student’s *t*-test. In bold are reported statistically significant values.

**Table 6:** Biochemical and metabolic parameters in adolescents from Groups A, B and C categorized into three sets according to the negative KIDMED items at T0 and T1.

KIDMED		SET 4			SET 5			SET 6		
**Subject Group A**		**(21 vs. 22)**			**(17 vs. 21)**			**(7 vs. 10)**		
**Parameters**		**Mean**	**SD**	***p*-Value**	**Mean**	**SD**	***p*-Value**	**Mean**	**SD**	***p*-Value**

Total cholesterol (mg/dl)	T0	160.38	25.18	**0.0014**	156.76	23.80	**0.0066**	161.14	20.71	**0.0024**
	T1	137.09	19.09		136.57	19.40		128.60	16.22	
LDL (mg/dl)	T0	94.62	28.96	**0.0052**	89.94	27.97	**0.0257**	91.71	29.47	**0.0251**
	T1	73.32	17.16		72.71	17.34		66.00	12.42	
HDL (mg/dl)	T0	53.67	12.68	0.6915	55.47	12.96	0.4449	57.57	16.28	0.3317
	T1	52.14	12.42		52.24	12.72		50.40	13.19	
Triglycerides (mg/dl)	T0	60.86	22.10	0.6942	57.41	20.05	0.9232	61.14	24.33	0.9703
	T1	58.18	22.20		58.10	22.75		60.70	23.31	
Glucose (mg/dl)	T0	83.29	6.20	**0.0052**	83.71	6.02	**0.0034**	82.43	4.58	0.0566
	T1	77.09	7.46		76.71	7.43		75.30	8.23	
Insulin (μIU/mL)	T0	12.66	6.28	0.2699	13.00	6.69	0.4122	10.94	3.43	0.1042
	T1	15.14	8.09		15.06	8.28		16.40	7.77	
**Subject Group B**		**(29 vs. 29)**			**(22 vs. 27)**			**(11 vs. 12)**		
**Parameters**		**Mean**	**SD**	***p*-Value**	**Mean**	**SD**	***p*-Value**	**Mean**	**SD**	***p*-Value**

Total cholesterol (mg/dl)	T0	155.41	30.90	**0.0454**	158.32	28.29	**0.0406**	168.64	26.09	**0.0264**
	T1	139.39	28.67		141.15	28.44		140.73	29.63	
LDL (mg/dl)	T0	81.79	24.43	**0.0437**	82.82	22.78	0.0714	87.09	19.80	**0.0473**
	T1	69.04	22.63		70.88	22.32		67.82	23.67	
HDL (mg/dl)	T0	58.93	16.51	0.9399	60.59	16.40	0.5877	66.27	17.98	0.4931
	T1	58.61	16.04		58.04	16.18		60.64	20.53	
Triglycerides (mg/dl)	T0	72.97	42.15	0.1542	74.09	44.48	0.2355	75.18	30.10	0.4009
	T1	59.04	30.35		61.19	30.44		62.64	39.01	
Glucose (mg/dl)	T0	81.97	8.29	0.2480	82.50	7.28	0.2710	78.73	6.12	0.6824
	T1	79.04	10.67		79.46	10.97		77.45	8.31	
Insulin (μIU/mL)	T0	9.11	4.23	0.7112	8.52	3.41	0.3300	8.05	3.52	0.3027
	T1	9.50	3.74		9.56	3.87		9.58	3.39	
**Subject Group C**		**(23 vs. 26)**			**(16 vs. 20)**			**(12 vs. 9)**		
**Parameters**		**Mean**	**SD**	***p*-Value**	**Mean**	**SD**	***p*-Value**	**Mean**	**SD**	***p*-Value**

Total cholesterol (mg/dl)	T0	146.26	23.22	0.6615	146.63	25.11	0.7159	140.33	21.80	0.9923
	T1	143.23	24.70		143.40	27.03		140.44	30.50	
LDL (mg/dl)	T0	72.22	21.45	0.7994	70.38	23.43	0.6938	65.17	14.90	0.6434
	T1	73.62	16.79		73.15	18.53		69.00	22.49	
HDL (mg/dl)	T0	62.09	12.64	0.3165	64.25	14.34	0.2951	63.33	14.60	0.4158
	T1	58.62	11.37		59.50	12.45		58.00	14.46	
Triglycerides (mg/dl)	T0	60.17	27.71	0.4246	59.88	31.40	0.4767	59.50	26.75	0.4517
	T1	54.54	21.15		53.55	21.24		67.67	19.89	
Glucose (mg/dl)	T0	86.96	4.78	**0.0001**	86.38	5.21	**0.0001**	86.58	5.12	**0.0054**
	T1	77.35	6.79		77.05	6.96		78.89	6.11	
Insulin (μIU/mL)	T0	10.78	3.04	**0.0274**	10.83	3.37	**0.0338**	10.63	3.65	0.3497
	T1	8.51	3.85		8.06	3.98		9.10	3.60	

LDL, low-density lipoprotein; HDL, high-density lipoprotein. Statistical differences were determined by Student’s *t*-test. In bold are reported statistically significant values.

## Data Availability

The datasets used and/or analyzed in the current study are available from the corresponding author upon reasonable request.
